# Advancement of the standard *in vivo* hand rub test methods: a critical comparison of the health care personnel handwash (ASTM E1174) and the hygiene handrub (EN1500)

**DOI:** 10.1186/1753-6561-5-S6-P269

**Published:** 2011-06-29

**Authors:** JW Arbogast, B Okeke, F Brill, T Bradley, A Fraise, M Wilkinson, D Macinga

**Affiliations:** 1 GOJO Industries, Inc., Akron, Ohio, USA; 2 GOJO Industries, Inc., Milton Keynes, UK; 3 Dr. Brill + Partner GmbH , Hamburg, Germany; 4 University Hospitals Birmingham NHS Trust, Birmingham, UK

## Introduction / objectives

Recognized standards for evaluating alcohol based hand rubs (ABHR) differ significantly in methodology and success criteria. Hand hygiene authorities including the WHO and U.S. CDC have recognized inherent weaknesses calling out the need for improved *in vivo* efficacy methods.

## Methods

The European Standard EN1500 (Hygienic Handrub), ASTM Standard E1174 (Health Care Personnel Handwash), and a recently approved ASTM standard, ASTM E2755-10, were critically compared based on the written protocol and empirical evidence (i.e. actual performance in practice).

## Results

All methods use gram-negative bacteria as marker organisms and the measured endpoint is reduction on hands after test article application. ASTM E2755 includes *Staph. aureus*, more representative of a hand transmitted pathogen in healthcare environments. Both EN1500 and E1174 have unrealistic hand contamination procedures which necessitate evaluation of unrealistic volumes of product at excessive contact times. E2755 employs a low volume contamination method for dry, relatively unsoiled hands, allowing ABHR evaluation at realistic volumes and contact times. Success criteria are significantly different: EN1500 uses an internal reference and ASTM methods use specific log reduction minimum endpoints.

## Conclusion

Neither method approximates actual healthcare worker ABHR usage. None has success criteria based on evidence of clinical benefit or prevention of pathogen transmission. A single, globally recognized *in vivo* efficacy standard would be of significant value to the infection prevention community and is a vision worth working towards.

## Disclosure of interest

J. Arbogast Employee of GOJO Industries, Inc., B. Okeke Employee of GOJO Industries, Inc., F. Brill Employee of Dr. Brill + Partner GmbH , T. Bradley: None declared, A. Fraise: None declared, M. Wilkinson: None declared, D. Macinga Employee of GOJO Industries, Inc.

